# Complication rates and outcomes stratified by treatment modalities in proximal humeral fractures: a systematic literature review from 1970–2009

**DOI:** 10.1186/1754-9493-7-34

**Published:** 2013-11-24

**Authors:** Alexander Tepass, Bernd Rolauffs, Kuno Weise, Sonja D Bahrs, Klaus Dietz, Christian Bahrs

**Affiliations:** 1 Department of Trauma and Reconstructive Surgery, Eberhard-Karls University, Tübingen, Germany; 2 Department of Medical Biometry, Eberhard-Karls University, Tübingen, Germany; 3 Department of Radiology, Eberhard-Karls University, Tübingen, Germany

**Keywords:** Systematic review, Proximal humeral fracture, Treatment, Complication, Shoulder fractures

## Abstract

**Background:**

The optimal treatment of complex, displaced proximal humeral fractures is controversial. A systematic literature review of the time period from 1970 to 2009 was conducted. The purpose was to evaluate the clinical success and complications of the available treatment modalities to determine specific treatment recommendations for the different fracture patterns.

**Methods:**

The databases (PubMed/EMBASE) were searched for the time period (01/1970–09/2009). Study quality, treatment modalities, classification, outcome scores and complications of 200 publications including 9377 patients were analyzed. Interventions were compared by analysis of variance with subsequent Tukey’s-test. Complication rates among methods were compared by using Pearson’s-chi-square-test and pairwise comparisons using Fisher’s-two-tailed-exact-test.

**Results:**

Hemiarthroplasty, angle-stable plate and non-operative treatment were used for 63% of the follow-up-patients. For 3- and 4-part fractures, patients with hemiarthroplasty [3-Part: 56.4 (lower/upper 95% confidence interval (CI): 43.3-68.7); 4-Part: 49.4 (CI: 42.2-56.7)] received a lower score than different surgical head-preserving methods such as ORIF [3-Part: 82.4 (CI: 76.6-86.9); 4-Part: 83.0 (CI:78.7-86.6)], intramedullary nailing [3-Part: 79.1 (CI:74.0-83.4)] or angle-stable plates [4-Part: 66.4 (CI: 59.7-72.4)].

The overall complication rate was 56%. The most common complications were fracture-displacement, malunion, humeral head necrosis and malreduction. The highest complication rates were documented for conventional plate and hemiarthroplasty and for AO-C, AO-A, for 3- and 4-part fractures. Only 25% of the data were reported with detailed classification results and the corresponding outcome scores.

**Discussion:**

Despite the large amount of patients included, it is difficult to determine adequate recommendations for the treatment of proximal humeral fractures because a relevant lack of follow-up data impaired subsequent analysis. For displaced 3- and 4-part fractures head-preserving therapy received better outcome scores than hemiarthroplasty. However, a higher number of complications occurred in more complex fractures and when hemiarthroplasty or conventional plate osteosynthesis was performed. Thus, when informing the patient for consent, both the clinical results and the possibly expected complications with a chosen treatment modality should be addressed.

## Introduction

Epidemiological research has demonstrated both an overall and an increase of displaced complex proximal humeral fractures that are treated in a clinical setting, affecting especially woman older than 60 years [1-7].

The Neer-[8] and AO-/OTA [9]-classifications are commonly used.

Minimally displaced fractures can successfully be treated non-operatively [6,8,10].

The treatment of displaced fractures ranges from non-operative treatment to a large number of surgical head-preserving procedures or prosthetic replacement [11,12].

A Cochrane review based on the few available randomized controlled trials (RCT’s) and therefore on a low number of included patients was not able to prove the superiority of surgical treatment of displaced proximal humeral fractures over non-operative treatment [13].

Despite this review, it seems that surgical treatment is chosen more generously in USA and central Europe [7,14,15]. However, there is a lack of evidence-based recommendations regarding the treatment of this fracture and in particular for the different fracture patterns, on the basis of clinical success and complication analysis.

Possible complications and problems occurring following treatment include e.g., non-anatomic- and loss of reduction, implant malposition/-loosening and -perforation, infection, impingement, hardware failure, humeral head necrosis, posttraumatic osteoarthritis, mal-/nonunion, joint stiffness, heterotopic ossification, rotator cuff lesion, prosthetic migration,- (sub-)luxation or -loosening [12,16-23].

Due to increasing numbers of displaced fractures with potential surgical treatment, patient safety and quality of care also need to be considered.

The purpose of the current patient-safety-based systematic review was: 1) to evaluate the reported clinical outcomes of operative and non-operative treatment of proximal humeral fractures between 1970 and 2009; 2) to evaluate the reported incidence of the complications following the treatment of these fractures; 3) to determine the feasibility of providing treatment recommendations based on fracture patterns, clinical success and complications analysis.

## Material and methods

Based on PRISMA guidelines [24] a systematic review was carried out in the databases (PubMed/EMBASE) with the help of a query created by PubMed [*tuberosity fracture OR proximal humeral fractures OR (humeral head AND fracture) OR (surgical neck AND fracture) OR (humeral shaft AND fracture) OR (shoulder joint AND fracture)*] for the time period (01/1970–09/2009). The specific single MeSH term was used for both PubMed and EMBASE. No search restrictions were used. Titles and abstracts were assessed independently by two reviewers (CB/AT) with respect to defined inclusion and exclusion criteria. Candidate articles were then included for full-text analysis and again checked for the mentioned inclusion and exclusion criteria. The given number is the specific exclusion reason after full-text analysis. (for details see Additional file 1: Table S1).

The full-text of all included studies was analyzed and references of the included studies and published systematic reviews [13,25-29] were searched for additional literature. In case of follow-up publications, the most recent work was included and in case of topic-related publications by the same authors, the study of better quality was chosen. The quality of included articles was evaluated with the help of SEQUES [30], MINORS [31] and Downs-checklist [32]. If controversial opinions on study-quality existed, a consensus was reached between the two reviewers (CB, AT) [33-35].

The following data was recorded:

Level of evidence I-IV, author, title, journal, publication-year, number of included- and follow-up patients, age, gender, results according to Neer-[8] and AO-classification [9] and duration of follow-up(FU).

The collected treatment modalities were subdivided into non-operative treatment, head preserving surgical treatment [minimally-invasive-osteosynthesis (intramedullary wiring, minimal osteosynthesis miscellaneous, closed reduction and percutaneous fixation (CRPF), open reduction and internal fixation (ORIF) with K-wire osteosynthesis, screw osteosynthesis, tension-band-wiring, helix wire), conventional plate, angle-stable plate, intramedullary nail and external fixator], hemiarthroplasty and reverse prosthesis.

Some studies reported results of multiple surgical treatment modalities or differing periods of immobilization in case of non-operative treatment. The results were documented separately according to the respective methods of treatment. The absolute results (mean, median and range) of the Constant-(n = 139), Neer-(n = 48), UCLA-(n = 11), DASH-(n = 23), ASES-(n = 14), Japanese-Orthopedic-Association-Shoulder-Score (JOA) (n = 3), Hawkins-Scale (n = 4), Hospital for Special Surgery-Shoulder-Assessment (HSS) (n = 2), Oxford-Shoulder-Questionnaire (OSS) (n = 8), Subjective-Shoulder-Value (SSV) (n = 3) and Simple-Shoulder-Test (SST) (n = 6) were recorded for the overall FU-group and– if mentioned– for the different fracture-groups according to the Neer-and AO-classifications.

The following complications were documented for the overall FU-group and for respective fracture-groups: pre-or postoperative nerval lesion, preoperative vascular injury, infection, humeral head necrosis, posttraumatic osteoarthritis, malreduction, fracture-displacement, mal-/nonunion, tuberosity displacement, tuberosity malunion/-lysis/-nonunion, incorrect implant position, implant/ screw loosening, primary/secondary screw perforation, implant failure, reflex sympathetic dystrophy, heterotopic ossification, rotator cuff lesion, impingement, prosthetic sub−/−luxation, prosthetic loosening/-migration, glenoid notching, revision surgery and implant removal.

For angle-stable implants primary screw perforation was only recorded if this was mentioned explicitly in the text. If secondary screw perforation was described without mentioning fracture-displacement, the number of secondary screw perforations was documented as fracture-displacement at the same time according to our definition. Malunion was also classified as malreduction if no fracture-displacement was reported. Furthermore, fracture-displacement was also documented as malunion if the latter was not mentioned in the studies explicitly. Prosthetic loosening was recorded only if prosthetic revision was required.

Radiological evidence of prosthetic loosening was not documented without clinical correlate. All complications except for preoperative vascular and nerval injuries were documented as total number of clinical-radiological complications.

The study was approved by the ethics committee of Eberhard-Karls-Universität Tübingen(project no. 22/2009 A).

### Statistics

For statistical analyses JMP10 (Cary, NC 27513, USA) was used. Continuous variables (duration of FU, age, score results) were summarized by their means and standard deviations if they were normally distributed and otherwise by their medians and ranges. For the nominal and the ordinal variables (complications, classification-groups) we reported frequency distributions as percentages.

All studies were analyzed with respect to the final results of their absolute scores. We chose the widely used Constant-Score as gold standard. For studies which used other shoulder scores in addition to the Constant-Score (e.g. UCLA, Neer) we recorded the Constant-Score. Based on previous studies [36-42] we used a linear regression between the logit transformed results of the DASH-Score, the UCLA-Score and the Neer-Score with the logit transformed Constant-Score. This was performed in accordance to previously published articles [43,44].

The therapeutic interventions were compared with regard to the score results by an analysis of variance with subsequent Tukey’s-test taking into account the number of fragments according to Neer- and the AO-classification. For the individual fracture-types (Neer-Parts, AO-Types A, B, C, Neer-Groups) we compared the incidence of complications with respect to the method of treatment. Firstly, we tested the global hypothesis of equal complication rates among the different treatment methods by using Pearson’s-chi-square test. If this global hypothesis was rejected after suitable Bonferroni-Holm-adjustment of the significance level, we performed pairwise comparisons of treatments using Fisher’s-two-tailed exact test [45]. For the global hypotheses we used 0.05 as the level of significance.

## Results

### Selection and evaluation of literature

**Figure 1:**
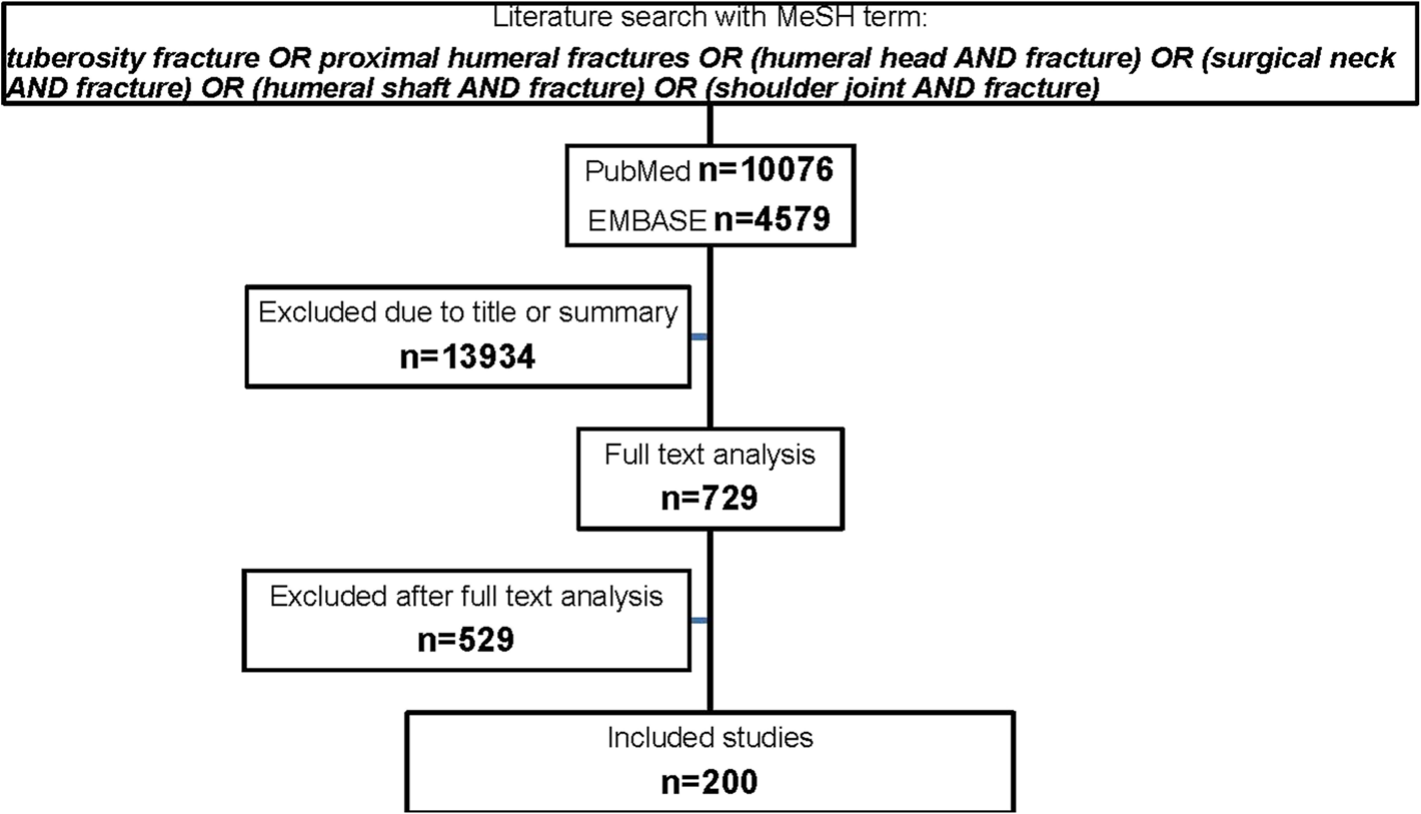
Flow-chart of literature inclusion/exclusion process.

The electronic literature research with PubMed resulted in 10076 and with EMBASE in 4579 studies. 671 publications (PubMed) and 58 (EMBASE) were selected for full-text review. Out of these, 200 studies were included (see Figure 1).

Included were 9 lesser-quality RCT’s or prospective comparative studies (4.5%), four (2%) case–control- or retrospective comparative studies and 187 (93.5%) case-series.

The average total quality score was 37.7% of the total achievable value. The highest mean percentage in all three evaluation scores was achieved by non-operative treatment in contrast to intramedullary wiring with the lowest (see Additional file 2: Table S2).

### Treatment

The 200 studies comprised 218 methods of treatment: 27 non-operative treatments, 16 CRPF, 15 intramedullary wiring, 25 ORIF, 5 minimal-osteosynthesis-miscellaneous, 20 conventional plate, 41 angle-stable plate, 18 intramedullary nail, 44 hemiarthroplasty, three reverse prosthesis and four external fixator.

### Patient characteristics

9377 of 13059 patients were followed-up (71.8%), 1033 patients (7.9%) died. The average patient age was 63.9 years (median 65, range 34–82 years, SD 8.8). On average, patients treated with hemiarthroplasty were older than patients who were treated non-operatively, with minimal-or plate osteosynthesis (p < 0.05) (see Additional file 3: Table S3).

### Follow-up and fracture classification

The mean follow-up was 30.5 months (median 24, range 3–156 months, SD 20.8).

Hemiarthroplasty (n = 1795), angle-stable plate (n = 2256) and non-operative treatment (n = 1831) were used for 62.7% of the FU-patients (see Additional file 3: Table S3). Treatment with hemiarthroplasty and ORIF was followed-up longer than angle-stable plate and intramedullary nail (p < 0.05).

The Neer-classification (2–4-parts) was used in 179 studies (89.5%), the Neer-Groups (I–VI) in 95 studies (47.5%) and the AO-classification (Types A, B, C) in 56 studies (28%). The majority of 2- and 3-part fractures were treated with angle-stable plates, whereas 4-part-, head-split-fractures and fracture-dislocations were treated predominantly with prosthesis. Nearly all Neer-Group-I fractures were treated non-operatively. Angle-stable plates were used for Neer-Group II-III or V fractures in contrast to Neer-Group-VI fractures, which were treated mainly with prosthesis. The AO-A fractures were treated predominantly non-operatively and in the case of AO-B and AO-C fractures with angle-stable plates (see Additional file 4: Table S4).

### Patient evaluation

The highest absolute Constant-Score (97.5 points) was documented for two patients with Group-II-fractures in one study (minimal-osteosynthesis-miscellaneous). The lowest absolute Constant-Score (40 points) was achieved following conventional plate osteosynthesis in one publication for AO-C-fractures (see Additional file 5: Table S5).

Additional file 5: Table S5 gives the total number of patients for which a Constant-Score was coded, and also the number of patients, for which a detailed fracture analysis was presented (as total number and as percentage). For example, in patients with a 3-part-fracture, only for 910 (40.9%) of 2227 patients a Constant-Score was reported. However, for those 910 patients with a reported Constant-Score, the mean score result was 70.7 points.

In summary, only for 4311 of a total of 11955 patients (36.1%) a detailed Neer-/AO-classification result was presented.

The highest mean Neer-Score (90.3 points) was documented for CRPF and 2-part-fractures with 8 patients in one publication. The lowest mean score (60.5 points) was described in three studies for 21 patients after conservative treatment of 4-part-fractures.

For Neer Group-II-fractures and head-split-fractures, no details were published on patient numbers, studies or absolute score. Patients with hemiarthroplasty received a mean of 82 points for 43 fractures in one publication.

Additional file 6: Table S6 gives the total number of patients for which a Neer-Score was coded, and also the number of patients, for which a detailed fracture analysis was given (as total number and as percentage). For example, in patients with a 2-part-fracture, only for 205 (12.6%) of 1626 patients a Neer-Score was reported. However, for those 205 patients with a reported Neer-Score, the score result was 83.5.

In summary, only 1643 of a total of 11844 patients (13.9%) were listed with a detailed Neer-/AO-classification (see Additional file 6: Table S6).

In general, apart from minimally invasive procedures (Min O Misc: best Constant score), and for hemiarthroplasty (worst score), the Constant-score results were in the same range. Comparable results were shown for the Neer-Score except that CRPF demonstrated the best and external fixator the worst Neer-score.

The statistical analysis showed that for 3- and 4-part-fractures, patients with hemiarthroplasty [3-part: 56.4 (lower/upper 95% confidence interval (CI): 43.3-68.7); 4-part: 49.4 (CI: 42.2-56.7)] received a lower score than different surgical head-preserving methods such as ORIF [3-part: 82.4 (CI:76.6-86.9); 4-part: 83.0 (CI:78.7-86.6)], intramedullary nailing [3-part: 79.1 (CI:74.0-83.4)] or angle-stable plates [4-part: 66.4 (CI: 59.7-72.4)] (p < 0.05). The Constant-values listed in this paragraph are recalculated out of the logit-transformed Constant-values that were used for further statistical analysis of the data. Therefore they differ from data shown in Additional files.

For the Neer-Groups III, V and VI no significant score differences between treatment modalities were found.

### Complications

Overall 5402 complications were reported in 200 studies. Out of these complications 158 (2.9%) were traumatic: 145 (91.8%) nerval lesions and 13 (8.2%) vascular injuries.

The overall postoperative clinical and radiological complication rate was 55.9%. Most common were fracture-displacement, malunion, humeral head necrosis and malreduction.

The highest complication rates were documented for conventional plate (87.3%) and hemiarthroplasty (67.8%). The lowest rates were found for non-operative therapy (29.6%) and for external fixator (42.9%) (see Additional file 7: Table S7).

The occurrence of specific complications was frequently reported in individual studies but not in all publications and the results often differed: e.g. traumatic vascular injury occurred in <9% [46] and traumatic nerval lesion in <20% [47].

Most common complications occurring after conservative treatment were: malunion (<89%) [48], fracture-displacement (<59%) [49], rotator cuff lesion (<51%) [50], posttraumatic osteoarthritis (<38%) [51], humeral head necrosis (<28%) [52], impingement (<24%) [51], nonunion (<12%) [53] and heterotopic ossification (<4%) [54].

Complications documented after surgical head-preserving therapy were: rotator cuff lesion (<100%) [55], malunion (<77%) [56], malreduction (<61%) [57], fracture-displacement (<54%) [58], humeral head necrosis (<50%) [59], impingement (<47%) [60], secondary screw perforation (<42%) [61], posttraumatic osteoarthritis (<33%) [60], screw loosening (<27%) [62], implant loosening (<27%) [63], nonunion (<27%) [63], reflex dystrophy (<25%) [59], heterotopic ossification (<24%) [64], infection (<23%) [65], implant failure (<14%) [66] and primary screw perforation (<14%) [67].

The complications listed for prostheses were: Glenoid notching (<94%) [68], tuberosity malunion/-lysis (<88%) [69], prosthetic migration (<71%) [70], prosthetic subluxation (<66%) [71], rotator cuff lesion (<64%) [72], heterotopic ossification (<54%) [73], posttraumatic osteoarthritis (<42%) [74], tuberosity displacement (<39%) [75] or -malreduction (<27%) [76], reflex sympathetic dystrophy (<21%) [77], implant failure (<19%) [78], infection (<18%) [46], tuberosity nonunion (<15%) [79], impingement (<15%) [80], prosthetic luxation (<11%) [81] and prosthetic loosening followed by implant revision (<6%) [82].

The rates of documented complications in total and split into subgroups differed considerably.

2080 complications (38.8%) were reported for separate fracture-subgroups. 1031 (49.6%) were documented with reference to Neer-Parts, 404 (19.4%) for the Neer-Groups and 585 (28.1%) for AO-Types. Accordingly most complications were reported for 3- and 4-part-fractures and for AO-Types-A/B/C.

Thus, no complications were reported for Neer-Group II. The highest rate was found for AO-C fractures (33.7%). The lowest rates for specific complications were found for preoperative vascular injury, incorrect implant position and prosthetic luxation (0%) and preoperative nerval lesion (3.4%). The highest complication rates were reported for tuberosity displacement (59.4%), humeral head necrosis (80.7%), nonunion (83.7%) and glenoid notching (93.8%) (see Additional file 8: Table S8). Complications were not represented unless they were listed specifically for the respective fracture-groups. Significant differences consisted in the occurrence of nonunion for 3-part-, humeral head necrosis for 4-part-, tuberosity displacement and fracture-displacement for A-fractures, impingement for B-fractures and in malreduction for B-and C-fractures (see Additional file 9: Table S9).

## Discussion

The purpose of this current patient-safety-based systematic review was to evaluate the clinical success and complications of the available treatment modalities of proximal humeral fractures to determine specific treatment recommendations based on different fracture patterns. Therefore 14.655 studies published between 1970 and 2009 were detected. Full-text analysis was conducted in 729 publications and 200 publications were included in this study.

Compared to other systematic reviews, we included a larger pool of data and also analyzed data for intramedullary nails and angle-stable plates. The three therapeutic options– hemiarthroplasty, angle-stable plate and non-operative treatment– accounted for 63% of the total data and for 50% of the relevant score-results. In general, when using the Constant- and Neer-Scores as parameters of functional outcome, the superiority of surgical over non-operative treatment has not yet been demonstrated in the current literature. However, this study demonstrated that the treatment of 3- and 4-part-fractures with hemiarthroplasty led to worse logit transformed Constant-Score results than head-preserving surgery.

CRPF and ORIF resulted in the highest Constant-Score but these values were based on only 2 studies [83,84]. The lowest average Constant-Score (53.7 points) was found for hemiarthroplasty. A possible explanation may be the high rate of 4-part-fractures, fracture-dislocations and head-split-fractures for which the clinical outcome is generally not as successful as for less complex fractures. Moreover, the findings demonstrated that the average age and accordingly the numbers of comorbidities were higher in the patients treated with hemiarthroplasty compared to other treatments [73,78,81,85-87]. Differences between various types and generations of prostheses and their influence on the union of the tuberosities may be another contributing factor [85].

Generally, it was difficult to compare the available systematic reviews in terms of the final score-results or the outcome because the methods that were used in the various reviews differed considerably (e.g. classification, inclusion criteria, study time, study aims, treatment procedures and statistical analysis). However, we henceforth present the results of other systematic reviews. Misra [26] analyzed 24 studies (3 comparative trials, 21 case series) on 3- and 4-part-fractures published between 1966–1999. Patients who had received a non-operative treatment suffered more pain than patients treated with head-preserving surgery or arthroplasty and they gained a lesser range of movement than patients treated with arthroplasty. The results were presented as percentage of excellent and good results. A Cochrane-review included 12 RCT’s published between 1966–2006 with 578 patients [13]. Conservative treatment was evaluated in 8 studies, 3 trials compared conservative and surgical treatment and 1 publication compared two surgical methods. Early physiotherapy adds to quick short-term recovery. Transcutaneous reduction and external fixation showed better restoration, safer healing and superior function compared to closed reduction for 2- to 4-part-fractures, but surgery was associated with more complications. Studies with small numbers of patients were included predominantly and angle-stable implants were not analyzed. Thus, no conclusions could be drawn due to insufficient evidence. The largest systematic review was presented by Lanting and co-workers [27]. They analyzed studies published between 1985–2004 and assessed patients who had received non-operative treatment, percutaneous fixation, intramedullary osteosynthesis, plate osteosynthesis and arthroplasty focusing on an ordinal-scaled Neer-Score. 66 studies with 2.155 patients were included (2 RCT, 3 comparative trials and 61 case-series). 2-part-fractures treated with plate fixation had a lower Neer-Score compared to intramedullary or miscellaneous-osteosynthesis. The treatment of 3-and 4-part-fractures with miscellaneous-osteosynthesis resulted in better outcomes and less pain compared to intramedullary-, percutaneous-, plate osteosynthesis or hemiarthroplasty. These results are- to some extent- comparable to the overall findings of our systematic review, which demonstrated that treatment of 3- and 4-part-fractures with hemiarthroplasty led to worse score results than head-preserving surgery. On the other hand our data does not support the superiority of head-preserving surgical treatment over conservative therapy, although a clear trend towards the operative treatment with angular-stable plates as therapy of choice was shown by two surveys [7,88].

Three authors who published implant-specific systematic reviews demonstrated score results which are also comparable to our findings. Nijs [29] and Kontakis [28] assessed hemiarthroplasty in 16 case-series (664/808 patients) published between 1998-2007/2008. The average Constant-Score was 53.9/56.6 points. An association between higher patient age and lower Constant-Score was presented and the importance of tuberosity union for the functional outcome was highlighted. Thanasas assessed angle-stable plates and included 12 publications up to 2007. The average Constant-Score was 74.3 points (76.9 points in 2-part-,75.8 points in 3-part-,67.6 points in 4-part-fractures) [25].

The second key finding of our present study was that the total complication rate after treatment reported in the 200 included studies was 56%. The highest rate was observed for AO-C, AO-A, for 4- and 3-part-fractures, as for conventional plate osteosynthesis and hemiarthroplasty when compared to non-operative treatment. However, statistical analysis showed for the respective classifications that in comparison to head-preserving surgical procedures, non-operative methods resulted in a higher rate of specific complications such as fracture-displacement and humeral head necrosis.

Generally it was also difficult to compare the available systematic reviews according to documented complications because the definitions and presentations differed considerably.

Misra [26] demonstrated that the infection rates of head-preserving procedures and arthroplasty were comparable. Handoll [13] reported that the infection rate was higher in external fixation and hemiarthroplasty in comparison to non-operative treatment. Anatomic reconstruction was less often achieved successfully by non-operative treatment than by head-preserving surgery [26]. On the other hand, treatment-failure, malreduction, humeral head necrosis and nonunion occurred more often during non-operative treatment compared to external fixation. The authors showed that revision rates of 4-part-fractures were higher for tension-band wiring than for hemiarthroplasty. Additionally, tension-band wiring led more frequently to infection, humeral head necrosis, nonunion, wire-perforation and osteoarthritis in comparison to non-operative treatment [13]. Lanting [27] documented complications of various treatments in relation to classification groups. Plate osteosynthesis for 2- and 3-part-fractures had the highest complication rates.

A systematic review of angle-stable plates (12 studies, 791 patients) listed the complication rates for infection (1.9%), nonunion (1.6%), humeral head necrosis (7.9%), hardware failure (0.7%), implant loosening (2.6%), displacement (12.2%), implant perforation (11.6%) and a revision rate of 13.7% [25].

Regarding study quality, a problem is undoubtedly the low number of cases which were presented in surgical RCT’s as well as indistinct presentations of the used methods [89-91]. Further problems of surgical RCT’s are blinding methods [89,92-95] and imbalance in the number of non-operative and surgical treatments [89,96]. However, methodically well-based observational studies are able to reduce bias and confounding despite the lack of randomization, and are able to deliver very valuable results [95]. Our experience was that some Evidence-IV-studies were outstanding in terms of prospective study design, number of FU-examinations and independent outcome assessors.

However due to the overall heterogeneity of our data no meta-analysis could be performed. Assigned reasons were due to the study conduction, e.g. the inclusion of a large number level IV evidence studies, the long time span with different implants and different implant generations, but also due to the poor presentation and large amount of missing data. A limitation of this study is that only 27% full-text analyzed articles were included in our review. The reasons for exclusion were– among others– missing absolute evaluation scores, publications in foreign languages and presentations as review articles. However, an absolute score result is eminently important to avoid methodological limitations such as the use of modified or non-validated scores, or insufficiently objective presentations of outcomes in categorized scales [97]. This present study design used an absolute score result to improve comparisons. This approach also led to the exclusion of studies and patients without data from which we could calculate absolute scores. Due to the fact that e.g. specific demographic and detailed presentations of outcomes according to the Neer-/AO-classification were often missing, the results have to be reviewed critically. Similar problems were reported by other authors [25,27].

Another critical point consisted in the lack of standard definitions and well-founded complication classifications in orthopedic literature. Complications are not only important for the evaluation of surgical safety, progress and improvement of treatment, but also to compare different implants for the same fracture pattern, and, last but not least, for the high-risk-patient identification [98]. Especially radiologically assessed complications such as malreduction, fracture-displacement, malunion or humeral head necrosis are not defined in satisfying ways.

We used our own definition concerning the complications especially, malreduction, fracture-displacement, malunion. This definition was done because of a common trias of complications after malreduction (especially in varus position) with consecutive secondary fracture displacement and secondary screw perforation [67,79,99-101].

The complication-rates reported in our study were higher than the rates reported in the literature. However, it has to be taken into account that only 39% of the complications could be associated with specific classification-groups.

Due to these limitations we present a level of evidence IV systematic review with introduction of bias and confounding factors.

In the field of trauma-related orthopedic surgery, the surgical techniques as well as the implants available have changed during the last decades. One other limitation of this study is that less modern techniques were compared to recent technical and implant developments. This is the case especially for ORIF and locking plates or intramedullary devices, and also for arthroplasty. Importantly, “modern” studies that were published within the last 3–4 years (e.g. recent studies that investigated locking plates) were not included. The time frame of this study (selected studies, time to publication) was the result of a large body of data, which had to be classified and re-allocated to treatment/complication- and classification-specific groups before any statistical analysis was possible. Overall, based on the included studies, we can’t rule out a publication bias.

In the future, prospective RCT’s, meta-analyses of individual data, or prospectively planned meta-analyses may be helpful to increase the data pool that will be available for analyses. Generally it has to be taken into account, that only validated scores and correctly applied outcome-scores should be used. It is also extremely important that possible complications- because of their large numbers- are clearly defined and correctly documented and presented. The clinical results and the complications that occur with a chosen treatment modality should be addressed clearly. This would not only improve study quality but it will also affect the safety and quality of care for the patient with a proximal humeral fracture.

## Conclusions

The publications that were analyzed suffered from a relevant lack of follow-up data due to moderate presentation quality. As a consequence, difficulties arose because consensus in the evaluation scores, defined standards and classifications of complications were missing. To unravel adequate treatment recommendations based on different fracture patterns was difficult. However, the treatment of 3- and 4-part-fractures with head-preserving surgery led to better score results than hemiarthroplasty. The superiority of head-preserving surgical treatment over conservative treatment was not supported by our data. The highest complication rates are to be expected for fractures that are treated more invasively (especially using hemiarthroplasty and conventional plates), and for AO-A-, AO-C-, and 3- and 4-part-fractures. A better stratification of subgroups, the adequate use of validated scores, and a clear definition and presentation of the occurring complications of a chosen treatment may lead in the future to the identification of the optimal management of specific fracture subgroups.

## Competing interests

The authors declare that there is no conflict of interest.

## Authors’ contributions

AT and CB contributed to acquisition of data, as well as analysis and interpretation of data. SDB, BR and KW were involved in drafting the manuscript with revisions critically and for important intellectual content. KD performed the statistical analysis and interpretation of the data. All authors read and approved the final manuscript.

## Supplementary Material

Additional file 1 Inclusion/exclusion criteria and numbers of excluded studies. Click here for file

Additional file 2 Mean and range of study-quality concerning different treatment modalities as percentage. Click here for file

Additional file 3 Demographic data of treatment modalities (number, mean and range). Click here for file

Additional file 4 **Number of fractures treated with different treatment modalities for different fracture-groups.** The percentage shows the proportion of total fractures in each fracture-group treated with the different modalities. N=number of fractures. 4-part-valgus impacted-fractures are presented additively, but are also included in the 4-part- group. The total of Neer Group-VI fractures are differentiated between fracture-dislocation and head-split-fractures. The total number of fracture-dislocation and head-split-fractures is lower than Neer Group-VI fractures because there was sometimes no specific presentation. The total number of FU-fractures presented in Additional file 4: Table S4 is higher than the total number of fractures in Additional file 3: Table S3 due to specific classification of fractures according to Neer-Parts/-Groups and AO-classification. Click here for file

Additional file 5 **Mean and range of the Constant-Score of different fracture-groups and their treatment modality.** N=number of fractures. The total number of fractures in FU is lower in Additional file 5: Table S5 than in Additional file 3: Table S3 because Additional file 5: Table S5 patients were specifically presented with regard to Neer-Parts/-Groups and AO-classification. In contrast, all patient data is shown in Additional file 3: Table S3 according to treatment modalities irrespective of classification. The reverse prosthesis and external fixator modalities are not presented in Additional file 5: Table S5 as the score fracture sub-specification was insufficient. Click here for file

Additional file 6 **Mean and range of the Neer-Score of different fracture-groups and their treatment modality.** N=number of fractures. Click here for file

Additional file 7 Total numbers of individual postoperative complications and their contribution as percentage to the total number of complications according to treatment modalities. Click here for file

Additional file 8 **Postoperative individual complications with reference to fracture-group.** The total number and the percentage of the ratio of individual complications and individual fracture-groups related to the FU-patients are listed. The second to last column presents the total of individual complications differentiated in the included publications. For comparison, the last column shows the complete total of all postoperative complications and the relation between the specific differentiated complications according to fracture-groups and the complete complications in percentage. Here, the total number of patients is higher than the total number of patients in Additional file 3: Table S3 followed-up due to specific classification of fractures according to Neer-Parts /-Groups and AO-classification. Click here for file

Additional file 9 **Statistically significant differences of complications presented by individual complication for significant different treatment modalities and fracture-groups with exact p-value.** > means first treatment modality is statistically worse in comparison to the second. < means first treatment modality is statistically better in comparison to the second. Click here for file
