# Supplementary figures and images for: Complication rates and outcomes stratified by treatment modalities in proximal humeral fractures: a systematic literature review from 1970–2009
Source: Patient Saf Surg. 2013 Nov 24;7:34. doi: 10.1186/1754-9493-7-34 (PMC4176190; doi:10.1186/1754-9493-7-34)

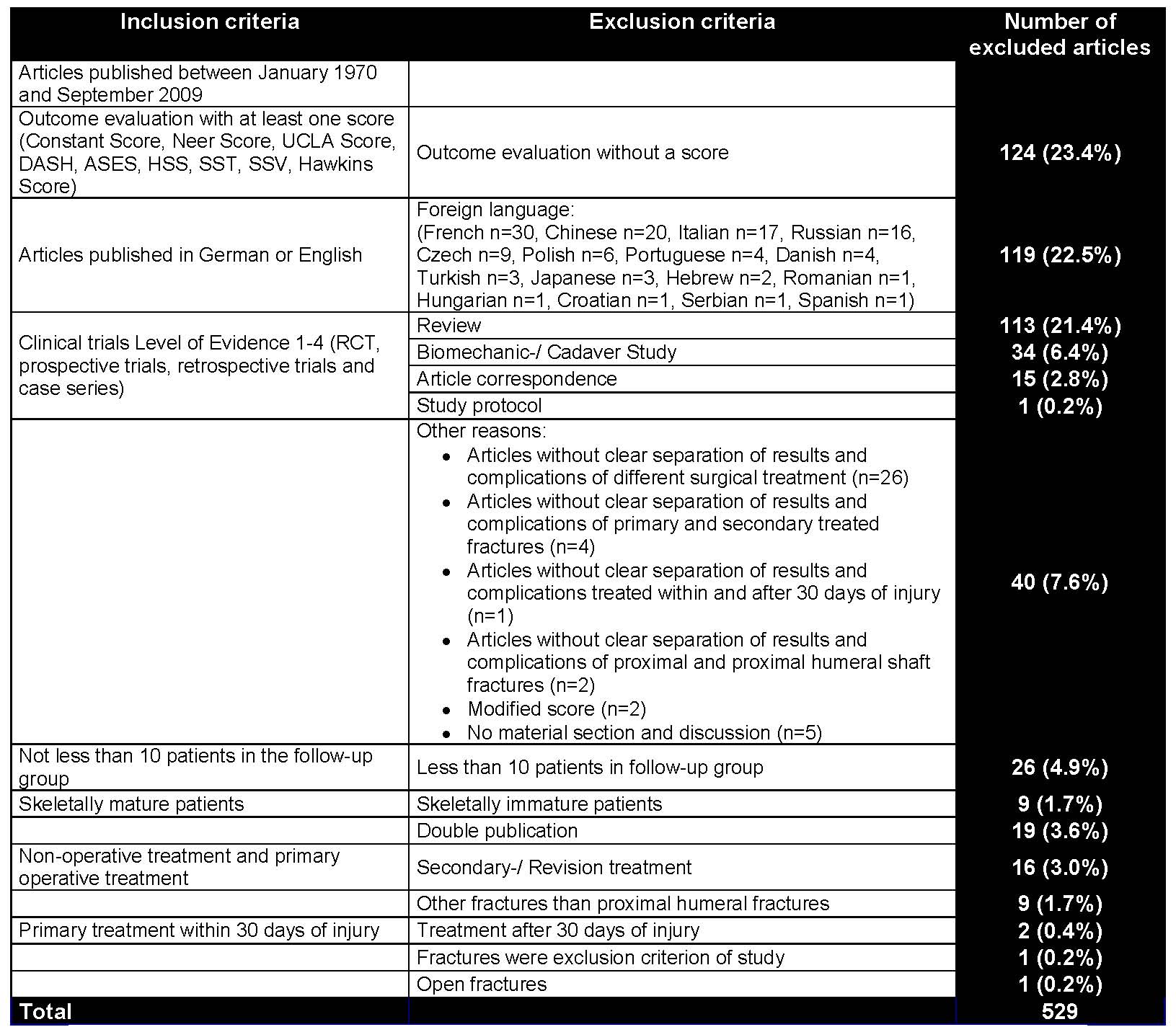

Supplement: Additional file 1 — Inclusion/exclusion criteria and numbers of excluded studies. [file 1754-9493-7-34-S1.tif]

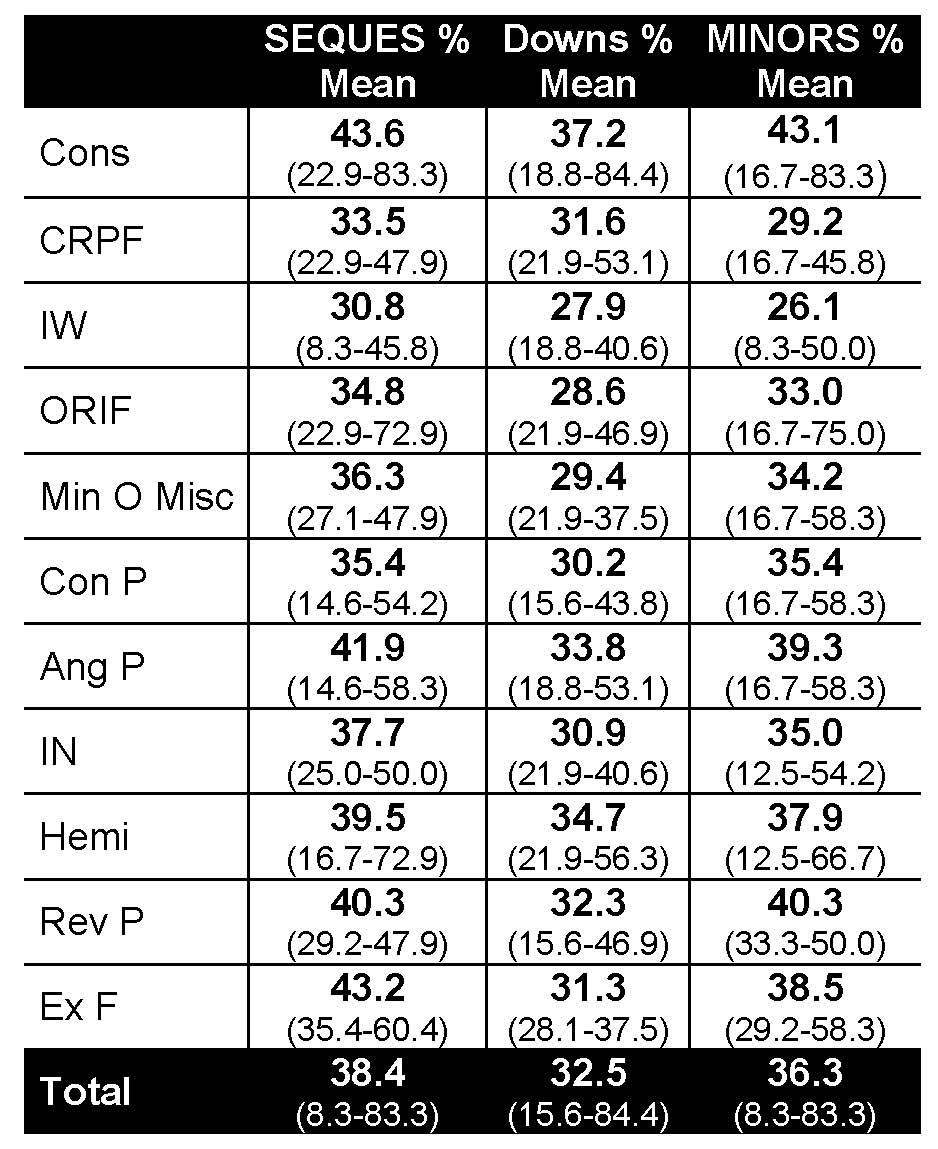

Supplement: Additional file 2 — Mean and range of study-quality concerning different treatment modalities as percentage. [file 1754-9493-7-34-S2.tif]

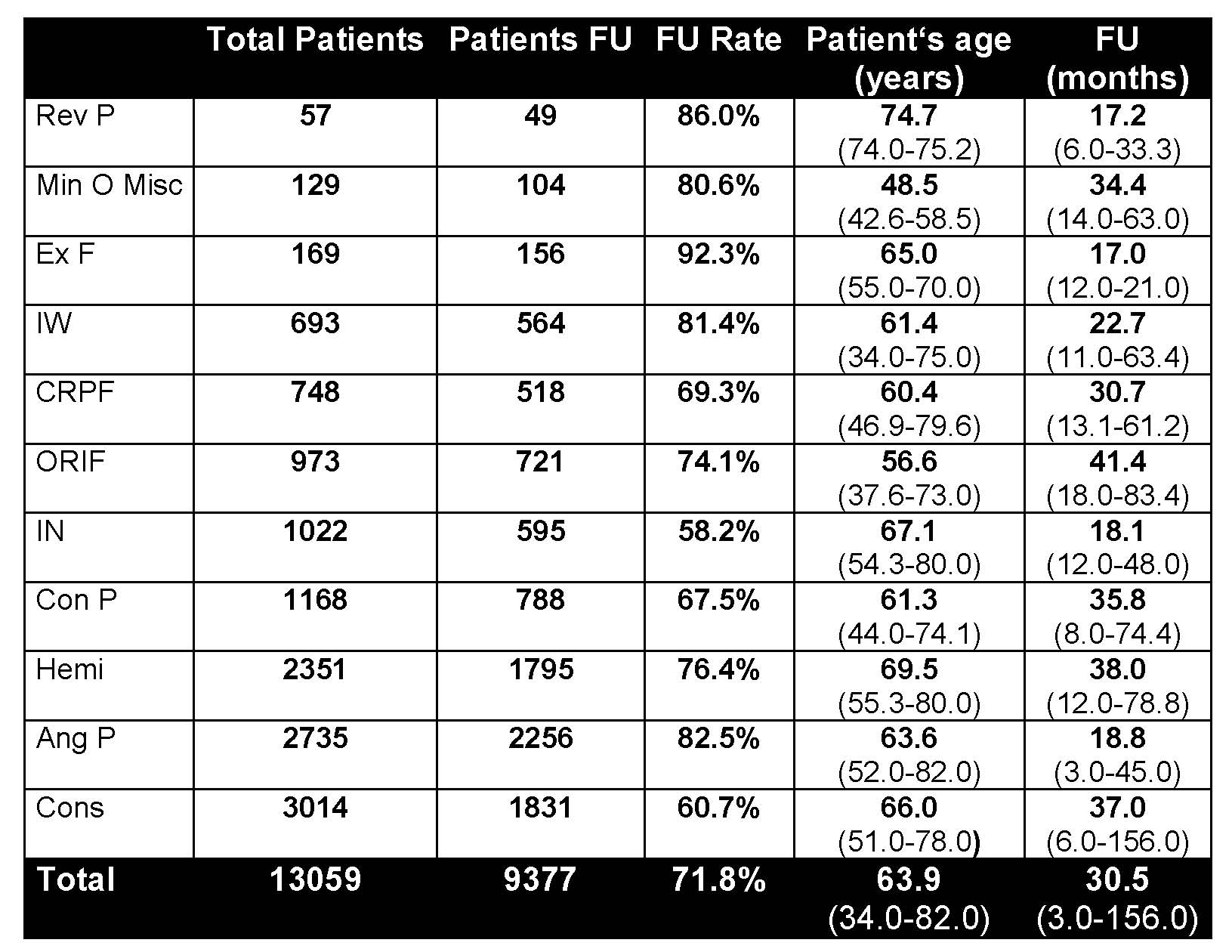

Supplement: Additional file 3 — Demographic data of treatment modalities (number, mean and range). [file 1754-9493-7-34-S3.tif]

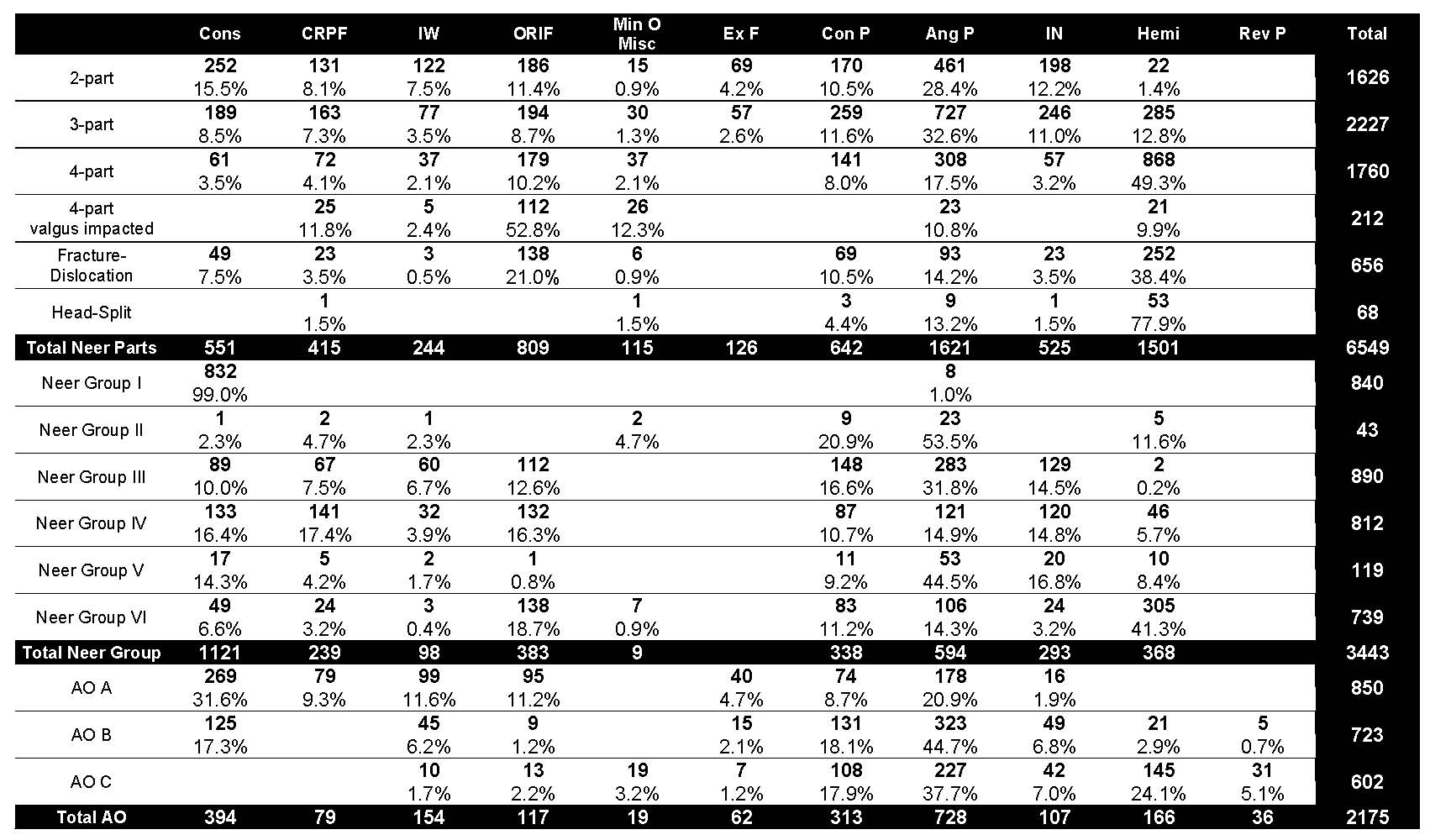

Supplement: Additional file 4 — Number of fractures treated with different treatment modalities for different fracture-groups. The percentage shows the proportion of total fractures in each fracture-group treated with the different modalities. N=number of fractures. 4-part-valgus impacted-fractures are presented additively, but are also included in the 4-part- group. The total of Neer Group-VI fractures are differentiated between fracture-dislocation and head-split-fractures. The total number of fracture-dislocation and head-split-fractures is lower than Neer Group-VI fractures because there was sometimes no specific presentation. The total number of FU-fractures presented in Additional file 4: Table S4 is higher than the total number of fractures in Additional file 3: Table S3 due to specific classification of fractures according to Neer-Parts/-Groups and AO-classification. [file 1754-9493-7-34-S4.tif]

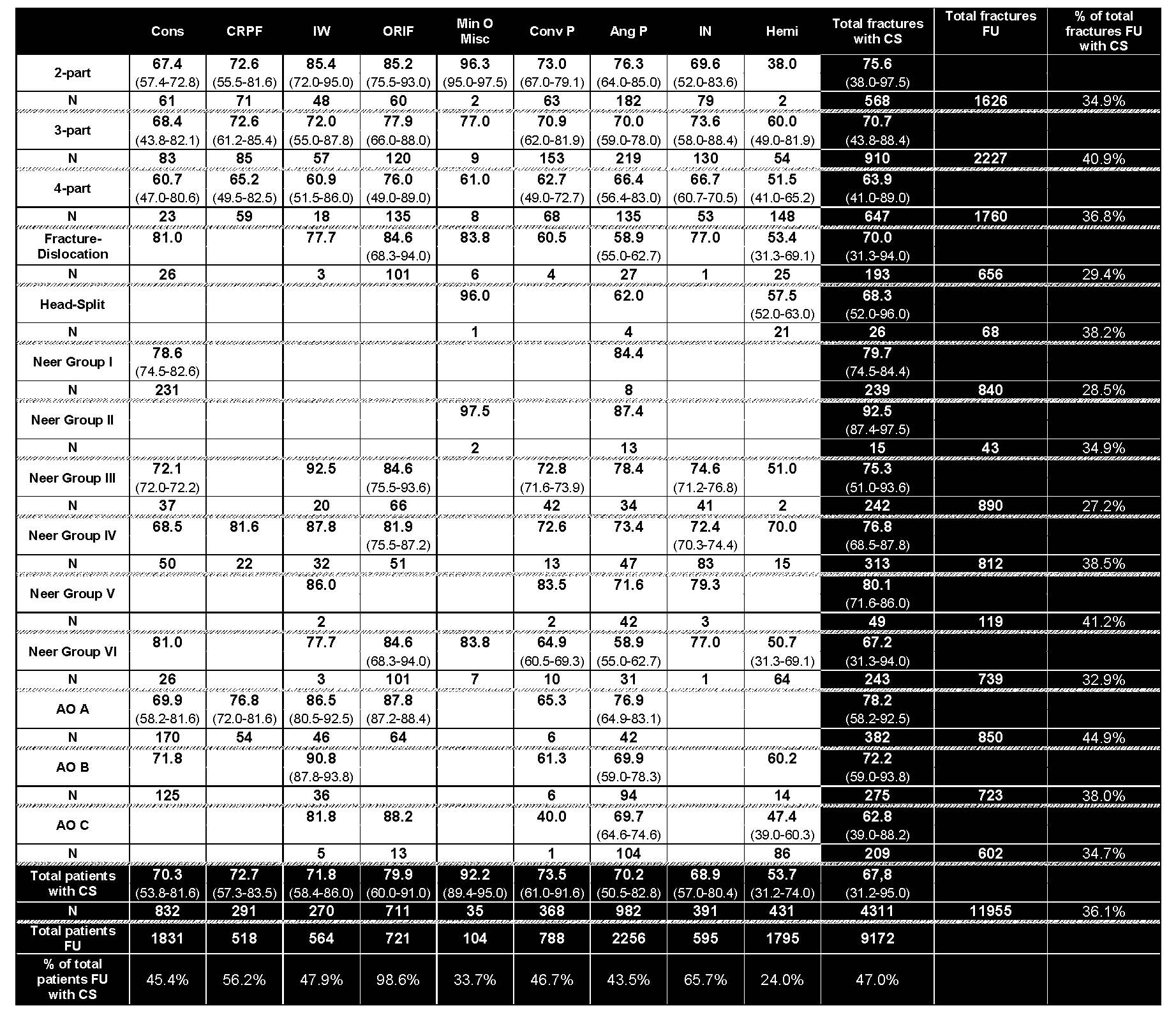

Supplement: Additional file 5 — Mean and range of the Constant-Score of different fracture-groups and their treatment modality. N=number of fractures. The total number of fractures in FU is lower in Additional file 5: Table S5 than in Additional file 3: Table S3 because Additional file 5: Table S5 patients were specifically presented with regard to Neer-Parts/-Groups and AO-classification. In contrast, all patient data is shown in Additional file 3: Table S3 according to treatment modalities irrespective of classification. The reverse prosthesis and external fixator modalities are not presented in Additional file 5: Table S5 as the score fracture sub-specification was insufficient. [file 1754-9493-7-34-S5.tif]

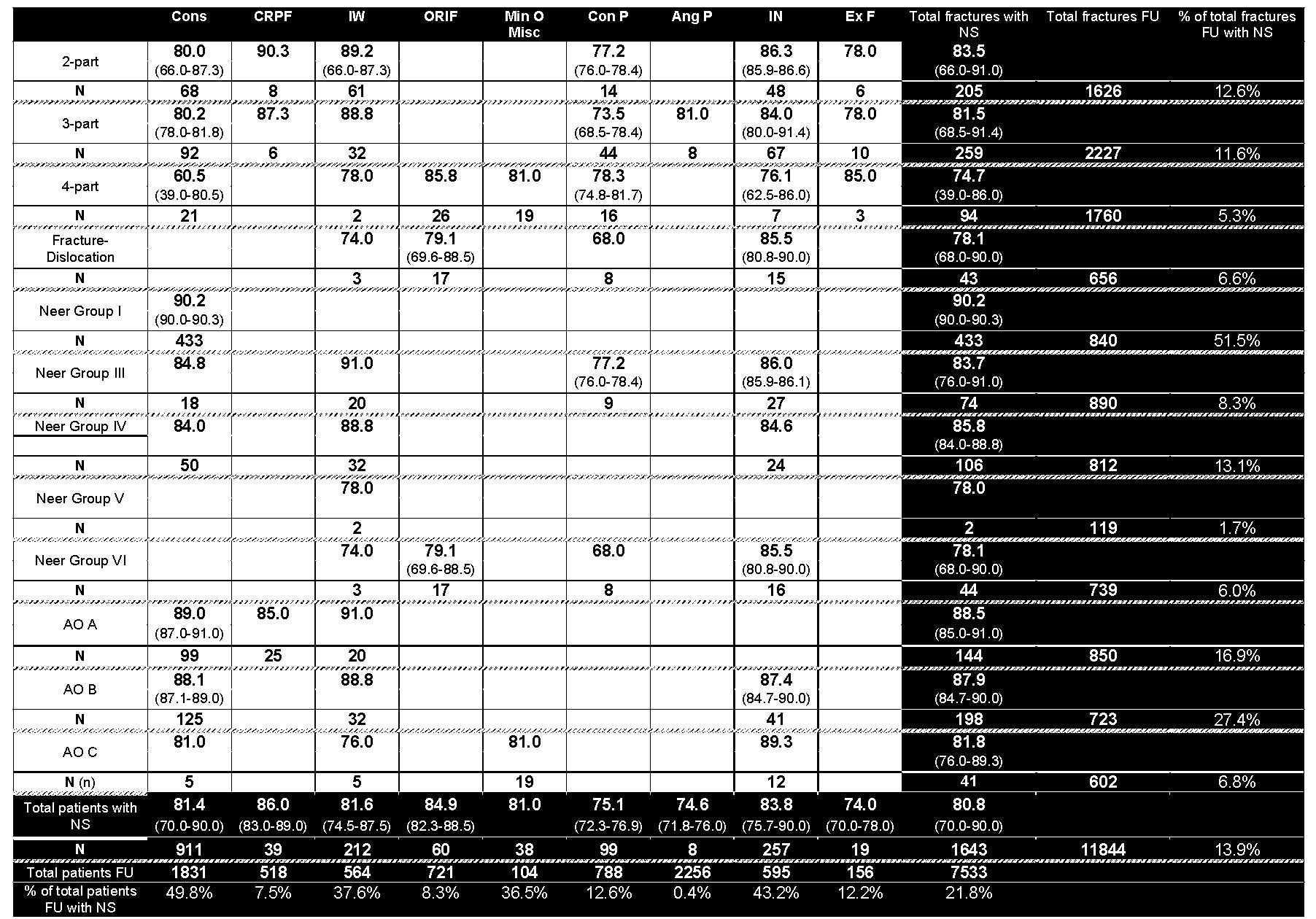

Supplement: Additional file 6 — Mean and range of the Neer-Score of different fracture-groups and their treatment modality. N=number of fractures. [file 1754-9493-7-34-S6.tif]

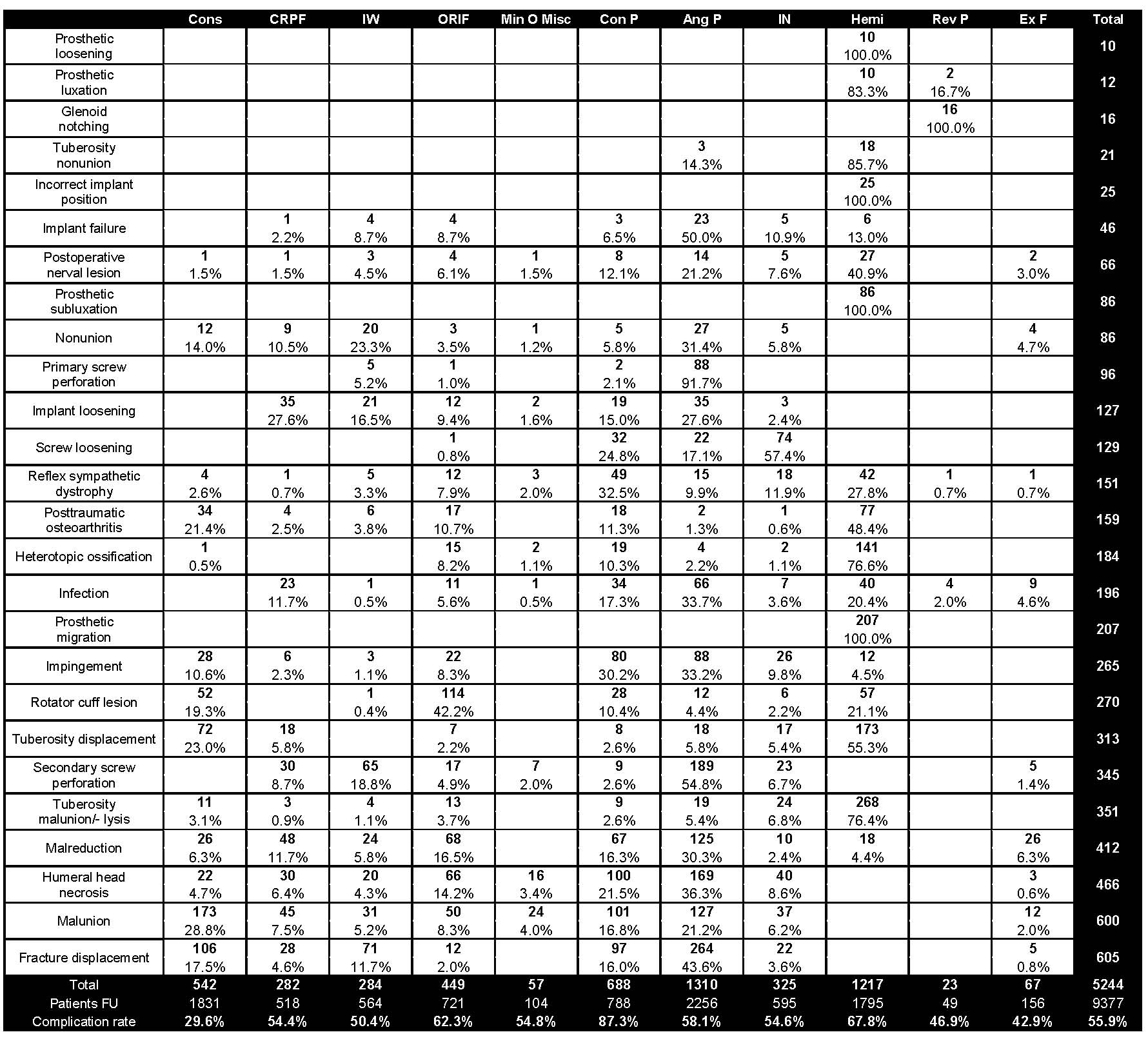

Supplement: Additional file 7 — Total numbers of individual postoperative complications and their contribution as percentage to the total number of complications according to treatment modalities. [file 1754-9493-7-34-S7.tif]

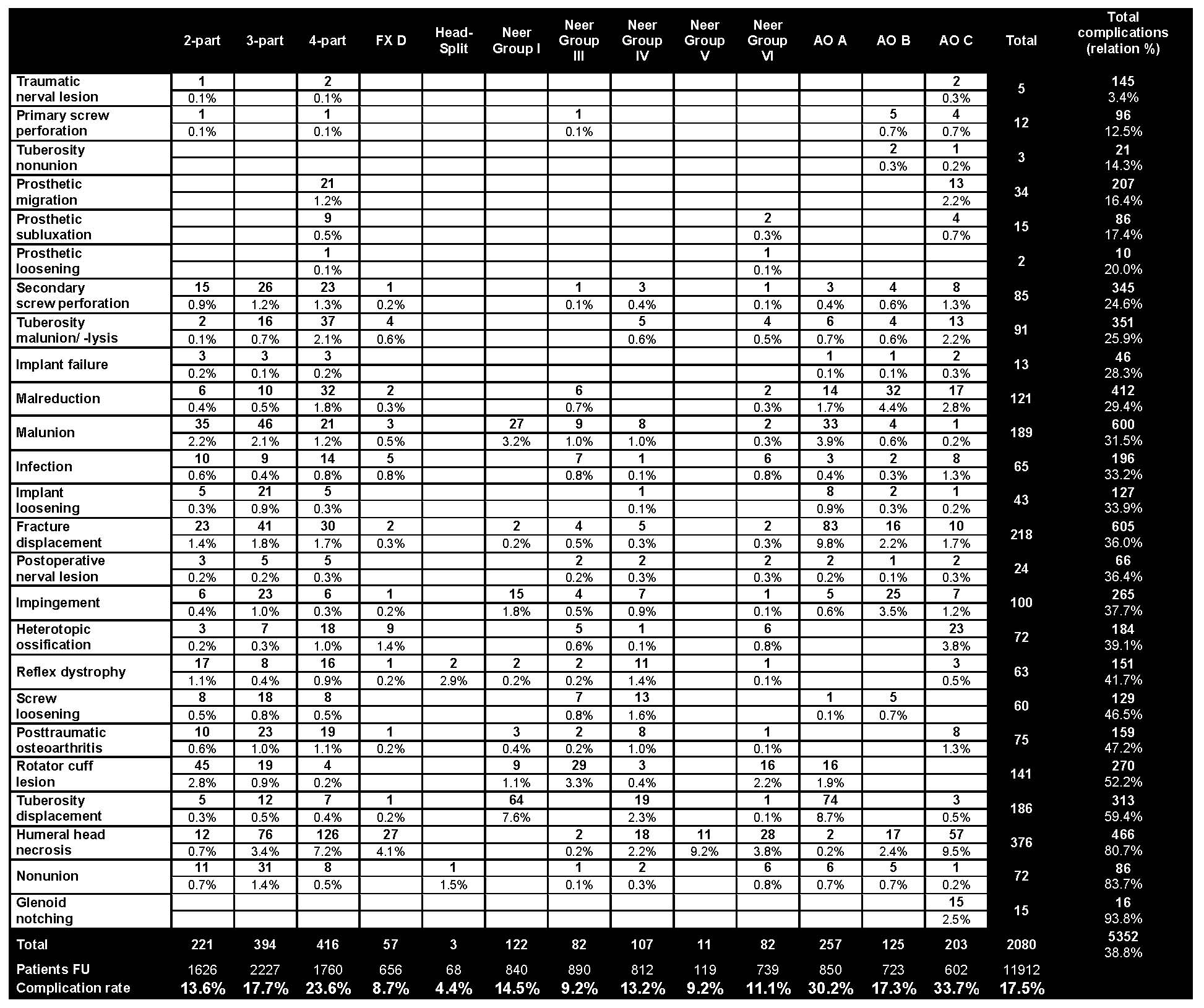

Supplement: Additional file 8 — Postoperative individual complications with reference to fracture-group. The total number and the percentage of the ratio of individual complications and individual fracture-groups related to the FU-patients are listed. The second to last column presents the total of individual complications differentiated in the included publications. For comparison, the last column shows the complete total of all postoperative complications and the relation between the specific differentiated complications according to fracture-groups and the complete complications in percentage. Here, the total number of patients is higher than the total number of patients in Additional file 3: Table S3 followed-up due to specific classification of fractures according to Neer-Parts /-Groups and AO-classification. [file 1754-9493-7-34-S8.tif]

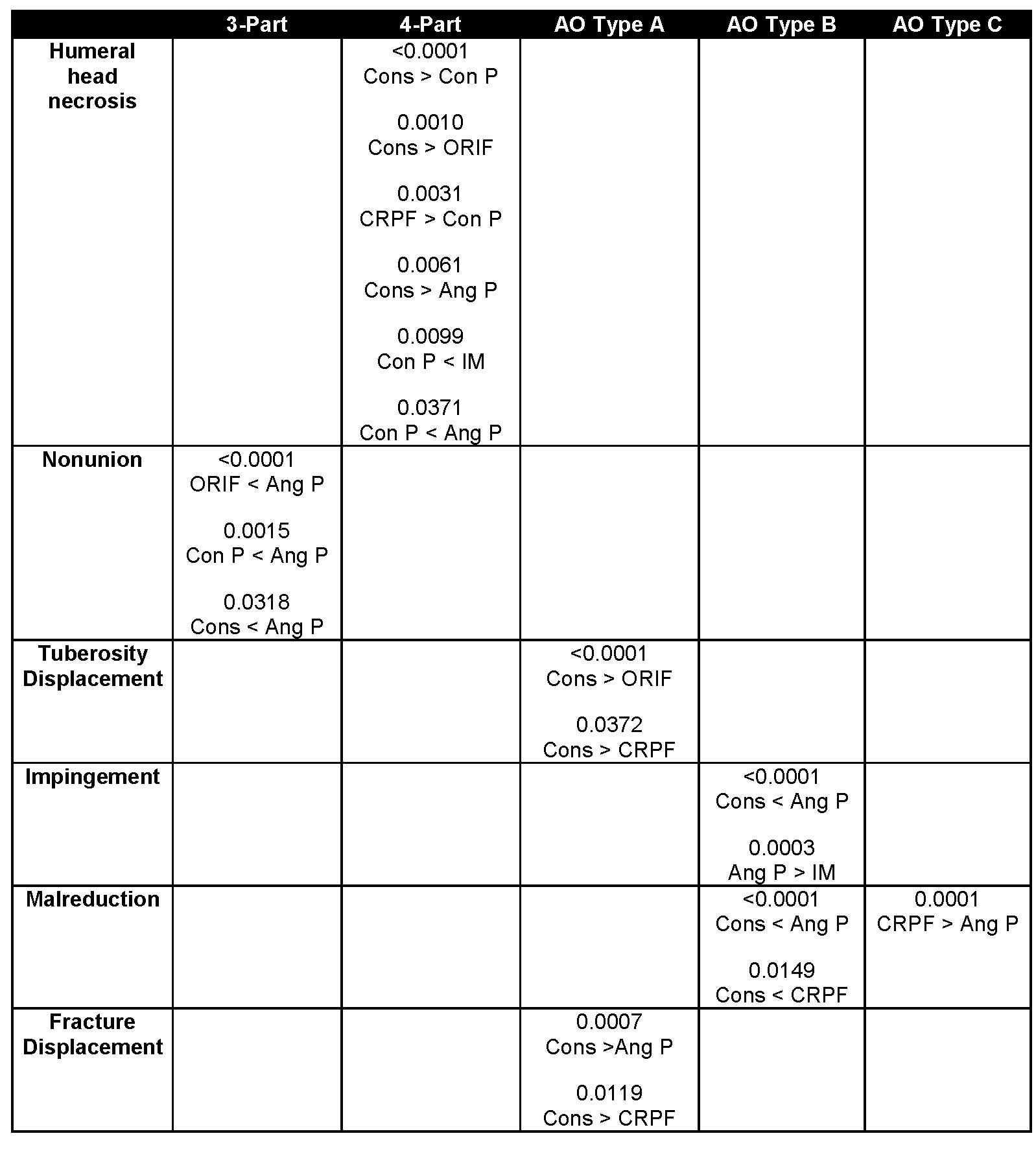

Supplement: Additional file 9 — Statistically significant differences of complications presented by individual complication for significant different treatment modalities and fracture-groups with exact p-value. > means first treatment modality is statistically worse in comparison to the second. < means first treatment modality is statistically better in comparison to the second. [file 1754-9493-7-34-S9.tif]
